# Acceptance of guidance to care at the emergency department following attempted suicide

**DOI:** 10.1186/s12888-017-1491-z

**Published:** 2017-09-13

**Authors:** W.P.H. Dekker, A.C.M. Vergouwen, M.C.A. Buster, A. Honig

**Affiliations:** 1 Department of Psychiatry/Research department, Onze Lieve Vrouwe Gasthuis-West, Jan Tooropstraat 164, 1061 AE, Amsterdam, the Netherlands; 2 Department of Epidemiology, Documentation and Health Promotion, Public Health Service Amsterdam, Nieuwe Achtergracht 100, 1018 WT, Amsterdam, the Netherlands; 3 Department of Psychiatry, VU University medical center, de Boelelaan 1118, 1007MB, Amsterdam, the Netherlands

**Keywords:** Attempted suicide, Guidance to care, Emergency department, Care utilization, Post-discharge care

## Abstract

**Background:**

Research, aimed at improving the continuity of care after hospital discharge following attempted suicide focuses on the effectiveness of the interventions. Little attention has been paid to patients who immediately decline guidance to advised post-discharge care. We aimed to identify differences between accepters and decliners of guidance to care (GtC) in relation to the characteristics of patients who presented at the emergency department (ED) of an urban hospital in the Netherlands after attempted suicide.

**Method:**

This cross-sectional study included all patients who presented at the ED of OLVG-West Amsterdam with a suicide attempt or intentional self-harm and were referred for psychiatric evaluation. Data were collected over a period of twenty months using a semi-structured questionnaire. Subgroups were described in relation the acceptance of GtC using univariate and multivariate logistic regression analyses.

**Results:**

In total, 257 patients were included. GtC was accepted by 77%. Suicide attempters who reported loneliness as reason for the attempt showed a positive relation to acceptance. No indication was found that patients at higher risk for suicide are more reluctant to accept GtC. Suicide attempters with a non-Western ethnicity, especially patients with a Turkish/Moroccan ethnicity, declined contact by the GtC nurse significantly more often. In addition, patients who currently did not receive care were significantly more often of non-Western ethnicity and younger than 25.

**Conclusion:**

Acceptance of GtC is high among patients who presented at the ED after attempted suicide. The patients who were the most reluctant to accept GtC were young suicide attempters of non-Western ethnicity who were not in current care. As this study is the first to address the acceptance of GtC, we point out two lines of inquiry for further research. First, reasons to accept or decline need to be investigated further since only interventions that are accepted by patients have a chance to improve clinically relevant outcome. Second, follow-up research is warranted comparing the adherence to advised post-discharge care and attempted or completed suicide among accepters versus decliners of GtC in various ethnic and sociodemographic subgroups.

## Background

Over 14,000 attempted suicides are seen annually at emergency departments (ED) in the Netherlands [1]. Such a high number of suicide attempts is worrisome, even more so in light of the fact that attempted suicide is an important risk factor for completed suicide: 50% of those who died by suicide made at least one previous attempt [2]. Furthermore, Dutch research shows that 40% of first time suicide attempters suffer from a recurrence [3]. High numbers of attempted suicides are especially worrisome if the rates of those receiving care fall behind. Among completed suicides, 58% did not receive mental healthcare prior to death [4]. Providing care for those that attempt suicide is important in preventing suicide, as unrecognized and/or untreated at-risk groups are more vulnerable to suicide [5]. Thus, the accessibility and acceptance of post-discharge care is important.

The Netherlands Mental Health Survey and Incidence Study-2 found that about a third of the people who attempted suicide had never sought help for personal problems [6]. The willingness to enter or re-enter mental healthcare facilities may be low due to negative experiences or fear of stigma. Research on barriers to care among suicidal people showed that for respondents who did not receive care in the past, the reasons for not seeking help were “low perceived need” and attitudinal barriers, such as the presence of stigma, low perceived efficacy of treatment, or the desire to handle the problem by themselves [7]. This underlines the importance of improving the accessibility and, equally important, the acceptability of care as part of suicide prevention strategies for those at risk for recurrent attempts [8]. Since a large proportion of those that attempt suicide become “visible” at the ED, this is where adequate care should be offered or provided to those at risk of recurrent suicide attempts.

The first task of psychiatric consultation at the ED is to provide medical care and to assess the severity of the suicide attempt. The second task is to assess the need for post-discharge care and to provide adequate referral, which should include providing clear advice for post-discharge care. However, in general hospitals, adequate referral to post-discharge care is not always guaranteed for patients who attempted suicide [9, 10]. Specifically, moments of “transfer” to advised post-discharge care are in need of improvement in order to ensure continuity of care [11]. In addition, the adherence to advised post-discharge care is known to be low among suicide attempters seen at the ED [12]. Therefore, an important part of suicide prevention is ensuring that advised care is accepted and adhered to.

Improving continuity of care following attempted suicide often involves outreach approaches after discharge from the hospital. Interventions aimed at a successful transition from in-patient to out-patient care for adult in-patients on psychiatric units are implemented at various stages: during the in-patient admission, in the early post-discharge period or during the transition from in-patient to out-patient care (i.e. bridging interventions) [13].

There is, however, a growing interest in brief interventions for patients presenting with self-harm or suicide attempts. Tools aimed at maintaining long-term contact and/or offering re-engagement with required services include letters, green cards, telephone calls and postcards. These interventions are distinct from other forms of outreach care and case management, as they do not include any formal therapy and only provide a minimal component of supportive intent or psychoeducation. Previous research has shown that these interventions significantly reduced the number of episodes of repeated self-harm or suicide attempts per person and seemed to have a positive, albeit non-significant effect in reducing repeated self-harm, suicide attempts and suicides [14]. Cedereke et al. reported on the effects of two telephone contacts, one at 4 months and one 8 months after discharge, in addition to usual care after a suicide attempt [15]. These contacts, which included motivational support to attend and/or continue treatment had some positive effects on treatment attendance in patients with treatment other than psychiatric care [15].

Based on the described research on brief interventions for patients presenting with self-harm or a suicide attempt, as well as on research on interventions aimed at successful transition from in-patient to out-patient care, we designed Guidance to Care (GtC). GtC aimes to overcome possible barriers to advised post-discharge care. GtC is offered before discharge from the ED of Onze Lieve Vrouwe Gasthuis West (OLVG-West) in Amsterdam as a form of personal assistance towards adhering to advised post-discharge care. It is offered in addition to the standard referral practices, and is carried out by the Public Health Service of Amsterdam [also see Methods section].

Contemporary evaluations of the above-mentioned interventions and outreach approaches focus mainly on the effectiveness of the interventions [16]. The goal of outreach is to contact every eligible patient after discharge [17–21]. Little attention has been paid to the identification of patients who immediately decline newly formed (outreach) approaches like GtC. Vaiva et al. noted that their “non-contactable” patients, those not reachable by phone, were less often depressed and fewer suffered from a somatic disorder [22]. Although these patients did not directly decline a form of outreach approach, they did refrain from this form of care. This supports the hypothesis that accepters of GtC differ from those that accept it.

Therefore, the aim of this study was to assess the acceptance of GtC among patients referred from the ED of OLVG-West (Amsterdam) to the department of Psychiatry for suicide assessment. In order to identify possible “at risk” groups, the acceptance of GtC was analysed in relation to demographic characteristics, including ethnicity, motives for the suicide attempt, risk factors for suicide, current care at the time of the attempt and on-site urine toxicology [4, 9, 10, 23].

## Method

### Guidance to care

To further improve the continuity of care by fostering compliance with advised post-discharge care in patients who attempted suicide, the department of psychiatry of the OLVG-West and the Public Health Service of Amsterdam initiated a pilot project in 2011. We targeted all patients at the ED presenting with attempted suicide, patients with suicidal ideation, and patients with deliberate self-harm. In line with current ED practice, the threshold for inclusion of suicide ideators was referral for psychiatric evaluation at the request of the ED physician. Patients with deliberate self-harm were included, because in this patient group the risk of suicide is higher than in the general population [24].

In our study we offered these patients, prior to discharge from the hospital, an intervention that was to take place in the early post-discharge period; more specifically, we offered GtC. This intervention was offered in addition to standard care which includes psychiatric evaluation and a suicide risk assessment based on on-site urine toxicology and on the Tool for Assessment of Suicide Risk (TASR) [2]. This tool was developed for health professionals to assess and manage suicidality based on epidemiology, risk factors and other known aspects of suicide. After psychiatric evaluation, recommendations and referral for post-discharge care were made. Standard procedure after discharge from OLVG-West includes informing the patient’s psychiatrist or general practitioner by telephone. The patient’s general practitioner also receives a letter of discharge. In the context of this pilot project, we offered the patients GtC after informing them of the recommendations and/or referral for post-discharge care.

Guidance to care is the process of providing support to individuals or groups to achieve a beneficial effect on adherence to post-discharge treatment advice. It was considered to be the preferred option for the patient that this support would be provided by someone who is not directly connected with the hospital nor to the advised post-discharge care organization, i.e. a third person in a neutral position. The Public Health Service of Amsterdam provided two community psychiatric nurses, they are nurse specialist in mental health care, who were assigned to this task. These GtC nurses had at least 10 years of experience working with psychiatric patients on an outpatient basis.

The GtC nurse functions as an intermediary between the patient and the advised post-discharge care. He/she checks whether the patient has adhered to the advised post-discharge care and, if not, stimulates and motivates the patient to do so. More specifically, by helping to remove various types of barriers (e.g. structural barriers by assistance during phone calls and organizing transportation or attitudinal barriers by identifying possible fears or stigma in order to provide reassurance). The GtC nurse contacted the patient within 10 days after discharge from the hospital to set up an appointment either by telephone or email.

The maximum duration of the GtC was six months, during which three face-to-face contacts (at home or in a public location) could be scheduled. Additional options were mail, text messages and/or telephone contacts, which were provided, if needed, at the discretion of the GtC nurse and the patient. No treatment was offered.

Patients were informed about the GtC before discharge from the ED, and leaflets with further information about the GtC were handed to the patients. Patients were then given the option to accept or decline this form of additional care. The protocol of the pilot was assessed by a medical ethics committee and was regarded as regular care; written informed consent was not deemed required. The efficacy of the pilot project is currently being investigated.

### Setting

This study was conducted at the 24/7 ED of the OLVG-West, a general teaching hospital located in Amsterdam (the Netherlands) that serves a multicultural urban population of about 140.000 inhabitants. About 50% of the population in the catchment area of this hospital is of non-Western ethnicity [25]. At the ED, about 300 patients per year are referred for acute psychiatric evaluation.

### Data collection

Data were collected over a period of twenty months, between April 2011 and December 2012. For this research, we designed a semi-structured interview based on literature and expert opinion (available upon request) [2]. Residents used paper notes, the semi-structured interview questionnaire and electronic data entry. Directly following the consultation and supervision of a staff member, the paper notes were included in the electronic patient file. The questionnaire included questions on sociodemographics, self-reported motives for the suicide attempt, ideation or self-harm, risk factors for suicide (TASR), current care, urine check and hospital admission after ED. Unlike past psychiatric diagnosis, de novo psychiatric diagnoses assessed in distressed patients on an ER are not reliable. Mental state assessment changes rapidly in time during and following ER visits. Therefore “a history of” or “current use of” psychiatric and/or addiction care was included in the dataset, but present diagnoses were not. All residents were trained by one of the authors (AH) in the use of the questionnaire, as well as in offering GtC on the ED. All residents worked under direct supervision of psychiatrists, two of whom where authors involved in this research.

Ethnicity was determined in accordance with the definition of the Statistics Netherlands (n.d.), which is based on the country of birth of the patient and of the patient’s parents [26]. According to this definition, anyone with at least one parent who was born in a foreign country is considered to belong to a “first-generation ethnic minority”, even though this person might hold the Dutch nationality. Ethnic minorities are further specified as either Western and non-Western minorities [26]. This distinction is based on the socio-economic and social-cultural status of countries. Non-Western counties are those belonging to the regions Africa, Latin-America, Asia (excluding Indonesia and Japan) and Turkey.

### Data analysis

Differences between decliners and accepters of GtC were determined with appropriate cross-tabulations (Chi-2) and logistic regression. Age, gender and variables with a univariate difference (*p*-value <0.10) were included in a two-step logistic regression. First, age and gender were forced into the model, and then variables that significantly improved the multivariate model were included by means of a Forward method. Differences were considered statistically significant if the likelihood ratio *p*-value was lower than 0.05. Uni- and multivariate Odds Ratios (OR) and 95% Confidence Intervals (95% CI) are shown. SPSS version 19 was used to analyse the data.

## Results

### Study population

Of all 257 patients referred by ED doctors for psychiatric evaluation of suicidality during the study period, the first referral was included in this study. Twenty-two of these patients were referred for suicide assessment more than once within the timeframe of this research (*n* = 28); these additional referrals were excluded. The study population included patients with suicide attempters or self-harm (*n* = 238) and suicide ideators (*n* = 19). Among those with a suicide attempt or self-harm, 218 patients (92%) were intoxicated, 19 (8%) had used a sharp object and 9 (4%) had used other methods. Eight patients with suicide attempts used multiple methods.

### Variables

#### Demographic characteristics

Most patients were female (64%); the average age was 39 years. Dutch ethnicity was most prevalent (46%). The largest non-white Dutch group was formed by Dutch-Turkish and Dutch-Moroccan patients (25%). The Turkish/Moroccan subgroup showed a higher proportion of female patients (78%) and of patients younger than 25 (43%). A lower level of education was found to be most common: 46% of patients indicated that their highest completed level was either basic or lower vocational education. Almost all patients were inhabitants of Amsterdam (91%), and 2% of the patients did not reside in the Netherlands. For more details, see Table 1.

**Table 1:** Description of study population and percentage of acceptance of guidance to care *n* = 257

Demographic characteristics	Number (%)	Acceptors Number (%)
*Total*	*257 (100%)*	*198 (77.1%)*
Gender		
Male	93 (36.2%)	76 (81.7%)
Female	164 (63.8%)	122 (74.4%)
Age		
< 25	55 (21.5%)	40 (72.2%)
25–44	111 (43.4%)	83 (74.8%)
45+	90 (35.2%)	75 (83.3%)
Ethnicity		
Dutch	116 (46.0%)	95 (81.9%)
Suriname & Dutch Antilles	23 (9.1%)	19 (82.6%)
Turkey & Morocco*^#^	63 (25.0%)	40 (63.5%)
Other Western	29 (11.5%)	24 (82.8%)
Other Non-Western	21 (7.8%)	16 (71.4%)
Highest completed education		
Lower education	113 (46.1%)	90 (79.6%)
Medium level of education	79 (32.2%)	59 (74.7%)
High level of education	53 (21.6%)	44 (83.0%)
Self-reported motives for the attempt (*multiple answers possible*)		
Loneliness*^#^	70 (27.2%)	62 (88.6%)#
Relational problems	108 (42%)	87 (80.6%)
Somatic problems	25 (9.7%)	22 (88.0%)
Financial problems	42 (16.3)	37 (88.1%)
Work related problems	40 (15.6%)	29 (72.5%)
Housing problems	17 (6.6%)	12 (70.6%)
Other	111 (43.2%)	82 (73.9%)
Risk factors		
Current problems considered unsolvable to patient	105 (42.0%)	84 (80.0%)
Suicide ideation	128 (50.2%)	105 (82.0%)
Suicidal intent	37 (14.6%)	30 (81.1%)
Suicidal plan	24 (9.5%)	21 (87.5%)
Family history	36 (14.4%)	31 (86.0%)
Recent substance abuse	102 (40.2%)	82 (80.4%)
Access to lethal means	125 (49.4%)	92 (73.6%)
Suicidal/violent command hallucinations^#^	24 (9.6%)	15 (62.5%)
Past suicide behaviour, *previous attempts:*		
0	106 (41.2%)	79 (74.5%)
1–2	82 (31.9%)	65 (79.3%)
3>	69 (26.8%)	54 (78.3%)
Current care at time of the attempt		
Currently receiving psychiatric/addiction care	150 (59.3%)	116 (77.3%)
History of psychiatric/addiction care (not currently)	54 (21.3%)	46 (85.2%)
Never received psychiatric/addiction care	49 (19.4%)	34 (69.4%)^#^
Hospital admission following ED^*#^	169 (65.8%)	142 (84.0%)

* *p* < 0.05, ^#^: included in logistic regression model (*p* < 0.10)

#### Risk factors and self-reported motives for the suicide attempt

Concerning risk factors for recurrent suicide attempt or fatal suicide, the majority 59% had a history of suicide attempt, 49% reported to have access to lethal means, 40% reported substance abuse and 10% mentioned imperative hallucinations.

Concerning the motives for the attempt, relational problems were reported most often (42%), followed by loneliness (27%). In about a third of the cases (39%), motives were related to other issues, such as work (16%), housing (7%) or financial problems (16%).

#### Current care

Over half of the patients were currently in psychiatric treatment (56%), and 12% currently received treatment for addiction. Accounting for dual treatment, the majority of patients received psychiatric care or treatment for addiction at the time of the attempt (60%). Of the patients without these forms of care (41%), 21% had previously received psychiatric or addiction care.

Patients who did not currently receive psychiatric and/or addiction care were significantly younger (*p* < 0.001). Of the patients younger than 25, 62% received no such treatment, versus 32% and 38% of the patients in the other age categories (see Table 1). Also, 55% of the patients of non-Western ethnicity received no psychiatric or addiction care, compared to 26% of the native Dutch patients (*p* < 0.001).

After psychiatric consultation at the ED, 44% of the patients were admitted to a somatic ward, 27% were transferred to the psychiatric unit, and 30% were discharged from the hospital.

### Acceptance of guidance to care

Overall, most patients accepted GtC (77%).

Table 1 shows the percentage of acceptances of GtC. Neither the age categories nor the gender categories showed significantly different proportions of acceptance. Patients of non-Western ethnicity declined GtC significantly more frequently than those of Western ethnicity (*p* < 0.05). Patients with a Turkish/Moroccan background showed the lowest acceptance percentage (64%).

Patients reporting loneliness as a trigger for the suicide attempt accept GtC significantly more frequently than those who did not (*p* < 0.01). Other self-reported motives were not significantly associated with differences in acceptance.

No significant variation in acceptance percentages was found when comparing the number of risk factors, nor was this the case for the hierarchically scaled risk factors. Patients reporting unsolvable problems as well as suicide ideation accepted GtC in 81% of the cases. For those who, in addition, mentioned suicidal intent, the percentage was 77%. Acceptance of GtC reached 88% among the patients who, in addition, had made suicide plans.

The univariate associations between individual characteristics and acceptance of GtC are shown in Table 1.

The overall acceptance percentage of GtC did not differ significantly between those patients who currently received psychiatric and/or addiction care and those who were not currently in treatment.

Significant differences in acceptance percentages were found between the patients who were and those who were not admitted to a somatic ward or a psychiatric unit after evaluation at the ED (84% versus 66%, *p* < 0.01).

Table 2 shows the results of the (forward) multivariate logistic regression analysis. Three variables were included in the equation: “Turkish or Moroccan ethnicity” (OR:0.4; 95%CI: 0.2–0.7), reporting loneliness as reason for suicidal behaviour (OR:2.7; 95%CI: 1.1–6.2), and hospital admission following ED (OR:3.7; 95%CI: 2.0–7.1). This analysis confirmed that the acceptance among the Dutch-Turkish and the Dutch-Moroccan patients remained significantly lower after adjustment for any of the other factors related to the acceptance of GtC.

**Table 2:** Logistic regression: acceptance of guidance to care facilitation

	Univariate analysis		Multivariate analysis		
	Odds Ratio	95% CI	Odds Ratio	95% CI	*p*-value
Gender (male vs female)	0.66	0.35–1.22	0.60	0.29–1.22	
Age (25–44 vs <25 years)	0.90	0.43–1.87	1.10	0.50–2.45	
Age (45+ vs <25 years)	0.53	0.24–1.20	0.60	0.24–1.51	
Hospital admission following ED	3.00	1.65–5.47	4.18	2.15–8.12	***
Turkish Moroccan background	0.40	0.21–0.74	0.39	0.19–0.83	*
Loneliness as a reason for TS	2.91	1.30–6.49	2.36	0.99–5.63	*
Suicidal/violent command hallucinations	2.29	0.94–5.55	–		
No (history of) psychiatric/addiction care	1.94	0.99–3.80	–		

*: p < 0.05, *** p < 0.001; Log likelihood ratiohood ratio; improvement of the model

## Conclusion and discussion

We investigated the acceptance of GtC and possible differences between accepters and decliners, based on patient characteristics.

### Overall acceptance of guidance to care

The overall acceptance of GtC was high, as 77% accepted immediately. Taking into consideration that 60% of the included patients were already receiving psychiatric care and/or treatment for addiction at the time of their attempt, this seems to be a high score which is in line with data from a Finnish study on healthcare contacts in relation to suicide attempts [27]. However, the perceived quality of the current treatment was not assessed, nor was the adherence to these treatments. Overall, the offer of GtC seems to have been appreciated, even though 23% of the patients declined immediately. Still, declining GtC cannot be interpreted as failing treatment adherence. GtC is not a form of treatment, and it is possible for patients to adhere to the advised post-discharge care without accepting GtC. We identified two groups who declined GtC significantly more often than others.

### Identified groups of patients that declined guidance to care more often

Our analysis showed that patients discharged immediately after treatment at the ED declined GtC significantly more often than those admitted to hospital. Hospital admission indicates either a more serious health problem and/or lack of a social support system that might provide social control of their behaviour at home. It is thus possible that these patients felt confirmed in their own “low perceived need” of care or that they felt that their problem or problems were not sufficiently acknowledged. Both lines of thought may give way to decline further care and need to be investigated further.

The second group of patients who declined GtC significantly more often, were patients with a non-Western ethnicity, especially the Dutch-Turkish and Dutch-Moroccan group. Acceptance among the Dutch-Turkish and Dutch-Moroccan group remained significantly lower even after adjustment for any of the other factors related to the acceptance of GtC. Further differentiation between the use of care and demographic characteristics showed that patients currently not receiving care were significantly more often of non-Western ethnicity and younger than 25 years of age. The Dutch-Turkish and Dutch-Moroccan patients constituted the largest ethnic minority group (25%), and 61% of this group received no psychiatric or addiction care at the time of the attempt. This corresponds with previous findings of healthcare use among Turkish and Moroccan youngsters in the Netherlands, which indicated that they are less likely to use mental health services [23, 28, 29].

The large number of ethnic minority patients seen on our ED may conceivably be related to the fact that about 50% of the population in the catchment area of our hospital is of non-Western ethnicity. Notwithstanding, others reported higher attempted suicide rates among young female minority groups, indicating a broader trend [30]. Paradoxically, however, the fatal suicide rate among females of Moroccan and Turkish ethnicity in the Netherlands is lower than average [31]. Nevertheless, there is cause for concern, given the lower coverage of treatment in this group combined with the lower proportion of acceptance of GtC found in the present study.

Declining GtC might be related to attitudinal barriers, such as shame, since the GtC involves “a third party”, the GtC nurse. Research on help seeking behaviour among adolescent girls from Turkish and Moroccan backgrounds showed that these minority groups more often mentioned “fear of negative reactions” as a barrier to seeking help than their Dutch counterparts [32]. Issues of anonymity and confidentiality were addressed in our study, as offering GtC always included the explanation that the GtC nurse was bound to confidentiality. This, however, seems not to have diminished the barrier to accepting GtC.

Another consideration might be the ethnicity of the GtC nurse. Both of the GtC nurses were of Dutch ethnicity. Although some authors indicated the lack of ethnically diverse staff as a point of improvement [33], focus group discussions reported more diversity in the preferences of young female ethnic minorities. Some felt that a professional with the same ethnic background would be better able to help, while others indicated that it did not matter to them, and yet others expressed concerns that a professional from the same ethnic background might have family relations within their Moroccan community [32].

### Differential rates of acceptance

We found no significant differences in acceptance solely based on age, despite the significantly higher proportion of younger patients without current psychiatric and/or addiction care. Furthermore, patients with a high-risk profile for further attempts and suicide did not decline more often than those at lower risk.

Those at highest risk for a recurrence of suicide attempt, e.g. patients with a large number of previous attempts, did not decline GtC more often. Over half of the patients seen at the ED reported a previous attempted suicide. Moreover, after the current attempt, half reported continued suicidal ideation. Such a high incidence of suicidal ideation just hours after the attempt is consistent with literature contesting the cathartic effect, i.e. the decrease in suicidal symptoms caused by the outward expression of suicidality via a suicide attempt [34, 35]. Research by Beautrais found higher rates of further attempts and higher rates of subsequent suicide among patients who had made medically serious attempts and failed to be relieved to have survived, still wanting to die, and stating the intention to make a further attempt [36]. On a positive note, we found that GtC was accepted by 88% of the patients in the top group of the hierarchically ordered risk factors, i.e. those who said to have made concrete plans for suicide.

One factor showed a significant positive relation with acceptance of GtC: patients who reported loneliness as the motive for the suicide attempt accepted GtC more often. As self-reported motives had to be expressed without probing, we belief that these patients felt assertive enough to not only admit loneliness, but also to accept help, as the prospect of personal contact might have appealed to them. This needs to be investigated further.

### Study limitations

This study is the first addressing the acceptance of GtC among patients presenting at the ED with self-harm, suicidal ideation or attempted suicide. In contrast to the offering after one month after the suicide attempt of two telephone contact at 4 and 8 months in the study of Cedereke et al. [16], our intervention was offered at the time of discharge from the hospital, and the GtC nurse would contact the patient within 10 days after discharge. GtC was offered as an add-on to usual care in a real-world setting, making the implementation feasible. Some limitations need to be mentioned. Firstly, this study was performed in a teaching hospital in Amsterdam, the Netherlands. Therefore, the results may not be generalizable to other settings, nor to other health care systems. Second, we could not investigate a possible relation between the acceptance of GtC and psychiatric diagnoses, since the acute setting of the ED, precludes appropriate psychiatric diagnosing other than the assessment of current symptomatology. Also, we did not assess the reasons for acceptance or non-acceptance, which may be indicated in order to improve the rate of acceptance. Finally, we investigated the acceptance of GtC, and consequently no conclusions can be drawn regarding the effects of accepting GtC on the patient’s adherence to the advised post-discharge care or on further suicide attempts or completed suicide. Only interventions that are accepted by patients have a chance to improve clinically relevant outcome.

### Implications of the results

At this stage, we cannot recommend implementation of GtC based on this study, despite the high acceptance after proposing GtC. Follow-up studies should compare accepters and decliners – in various (ethnic) subgroups - regarding relevant outcome measures, e.g. adherence to advised post-discharge care, suicide attempts and suicide. In addition, reasons for accepting or declining GtC need to be investigated.

## Abbreviations

ED-Emergency Department: GtC-Guidance to Care
